# RRMSE-enhanced weighted voting regressor for improved ensemble regression

**DOI:** 10.1371/journal.pone.0319515

**Published:** 2025-03-17

**Authors:** Shikun Chen, Wenlong Zheng

**Affiliations:** College of Finance and Information, Ningbo University of Finance & Economics, Ningbo, China; National Institute of Electronics and Information Technology, INDIA

## Abstract

Ensemble regression methods are widely used to improve prediction accuracy by combining multiple regression models, especially when dealing with continuous numerical targets. However, most ensemble voting regressors use equal weights for each base model’s predictions, which can limit their effectiveness, particularly when there is no specific domain knowledge to guide the weighting. This uniform weighting approach doesn’t consider that some models may perform better than others on different datasets, leaving room for improvement in optimizing ensemble performance. To overcome this limitation, we propose the RRMSE (Relative Root Mean Square Error) Voting Regressor, a new ensemble regression technique that assigns weights to each base model based on their relative error rates. By using an RRMSE-based weighting function, our method gives more importance to models that demonstrate higher accuracy, thereby enhancing the overall prediction quality. We tested the RRMSE Voting Regressor on six popular regression datasets and compared its performance with several state-of-the-art ensemble regression algorithms. The results show that the RRMSE Voting Regressor consistently achieves lower prediction errors than existing methods across all tested datasets. This improvement highlights the effectiveness of using relative error metrics for weighting in ensemble models. Our approach not only fills a gap in current ensemble regression techniques but also provides a reliable and adaptable method for boosting prediction performance in various machine learning tasks. By leveraging the strengths of individual models through smart weighting, the RRMSE Voting Regressor offers a significant advancement in the field of ensemble learning.

## 1. Introduction

Ensemble learning is a powerful machine learning paradigm that combines multiple models, often referred to as “base learners,” to solve the same predictive problem. This approach is applicable to both classification and regression tasks, where the goal is to predict categorical labels or continuous numerical values, respectively. The fundamental idea behind ensemble learning is that by aggregating the strengths of diverse models, the ensemble can achieve higher accuracy and greater robustness than any individual base learner alone [1–3].

Formally, an ensemble  is defined as a collection of predictors aimed at approximating a target function *f*. Each predictor in the ensemble, denoted as $\hat{f}$, represents a different hypothesis or model trained on the same dataset. Mathematically, the ensemble can be expressed as:


$$F=\left\{\hat{f_{i}},i=1,\ldots ,k\right\}\;.$$ (1)

Here, *k* represents the number of base learners in the ensemble. The goal is to combine these base learners in a manner that leverages their individual strengths while mitigating their weaknesses. The final prediction of the ensemble, $\hat{f_{f}}$, is typically obtained by aggregating the outputs of the base learners. This aggregation can be achieved through various methods, such as averaging in regression tasks or majority voting in classification tasks.

The ensemble learning process generally involves three main steps [4,5]. The first step is ensemble generation, which entails selecting and training the base learners to be included in the ensemble. Depending on whether the same algorithm is used to generate all base learners, ensembles can be categorized as homogeneous or heterogeneous. A homogeneous ensemble consists of base learners that are all the same type, such as multiple decision trees trained with different subsets of the data [6]. In contrast, a heterogeneous ensemble includes base learners of different types, such as combining decision trees, neural networks, and support vector machines within a single ensemble [7].

The second step is ensemble pruning, where the ensemble is refined by removing some of the base learners. This step aims to eliminate models that contribute little to the overall performance, thereby reducing computational complexity and avoiding overfitting. Effective pruning strategies can enhance the efficiency and effectiveness of the ensemble by focusing on the most promising models. The final step is ensemble integration, which involves combining the predictions of the remaining base learners to produce the final output. For regression problems, this integration is typically performed using a linear combination of the individual predictions:


$$\hat{f_{f}}\left(x\right)=\sum_{i=1}^{k}h_{i}\left(x\right)\cdot \hat{f_{i}}\left(x\right)\;.$$ (2)

Here, *h*_*i*_ ( *x* )  represents the weighting function assigned to the *i* -th base learner’s prediction. The choice of weighting functions $h_{i}\left(x\right)$ is crucial, as it determines the influence each base learner has on the final prediction. Ensemble integration strategies can be broadly classified into two categories: constant weighting functions [8], where $h_{i}\left(x\right)$ are fixed constants, and non-constant weighting functions [8], where the weights vary depending on the input features *x* [9].

Despite the proven advantages of ensemble learning, a significant challenge remains in effectively determining the appropriate weights for combining base learners, especially in scenarios lacking domain-specific knowledge. Typically, ensemble voting regressors employ uniform weights, assigning equal importance to all base models regardless of their individual performance. This uniform weighting can limit the potential improvements in prediction accuracy, as it fails to account for the varying reliability and strengths of different models across diverse datasets [10].

To address this limitation, this study introduces the RRMSE (Relative Root Mean Square Error) Voting Regressor, a novel ensemble regression technique that leverages RRMSE to dynamically assign weights to each base model. By using RRMSE as the basis for weighting, the proposed method prioritizes models that exhibit lower relative errors, thereby enhancing the overall prediction quality of the ensemble. This approach provides a systematic and data-driven mechanism for weight assignment, even in the absence of extensive domain knowledge, thereby bridging the existing gap in weighted ensemble regression methods.

To evaluate the effectiveness of the RRMSE Voting Regressor, comprehensive experiments are conducted on six widely recognized regression datasets. These experiments compared the performance of the proposed method against several state-of-the-art ensemble regression algorithms, including traditional uniform-weighted ensembles and other advanced weighted approaches. The results demonstrate that the RRMSE Voting Regressor consistently achieves lower prediction errors across all tested datasets, highlighting its superior ability to enhance ensemble performance through intelligent weighting. This approach not only improves prediction accuracy but also contributes to the broader field of ensemble learning by offering a reliable and adaptable method for boosting predictive performance in various machine learning applications.

## 2. Ensemble learning for regression

Ensemble learning is a widely adopted approach in machine learning that aims to improve the performance of predictive models by combining multiple base learners. In regression tasks, ensemble methods are particularly useful for enhancing prediction accuracy and robustness by leveraging the strengths of diverse models. Ensemble methods for regression can be broadly classified into two main categories: averaging methods and boosting methods. This study focuses on averaging methods, specifically on developing a weighting function for heterogeneous ensemble models. Consequently, only the common averaging methods are discussed in detail below.

### 2.1 Averaging methods

Averaging methods are fundamental ensemble techniques that involve training multiple base learners independently and then combining their predictions by averaging. The primary advantage of averaging is its simplicity and effectiveness in reducing the variance of individual models, leading to more stable and accurate predictions. Averaging methods can be further divided into various approaches, each with its unique way of generating and combining base learners.

#### 2.1.1 Bagging regression.

Bagging, short for Bootstrap Aggregating [11], is one of the most popular averaging methods in ensemble learning. It aims to reduce the variance of base learners, thereby enhancing the overall stability and accuracy of the ensemble. Bagging achieves this by creating multiple subsets of the original training dataset through bootstrapping, which involves random sampling with replacement.

Mathematically, let $D={\left\{\left({\text{x}}_{\text{j}},{\text{y}}_{\text{j}}\right)\right\}}_{\text{j}=1}^{\text{n}}$ denote the original training dataset, where *x*_*j*_represents the feature vector and *y*_*j*_ is the target variable. Bagging generates *k* bootstrap samples $D_{i}$ for $\left(\text{i }=1,\ldots ,\text{k }\right)$, each of size *n*, by sampling with replacement from *D*. For each bootstrap sample $D_{i}$, a base regression model $\hat{{\text{f}}_{\text{i}}}$ is trained independently.

The ensemble prediction $\hat{{\text{f}}_{\text{b}}}\left(\text{x}\right)$ for a new input *x* is then obtained by averaging the predictions of all base learners:


$$\hat{f_{\text{b}}}\left(x\right)=\frac{1}{k}\overset{k}{\underset{i=1}{\sum }}\hat{f_{i}}\left(x\right)$$ (3)

**Fig 1:**
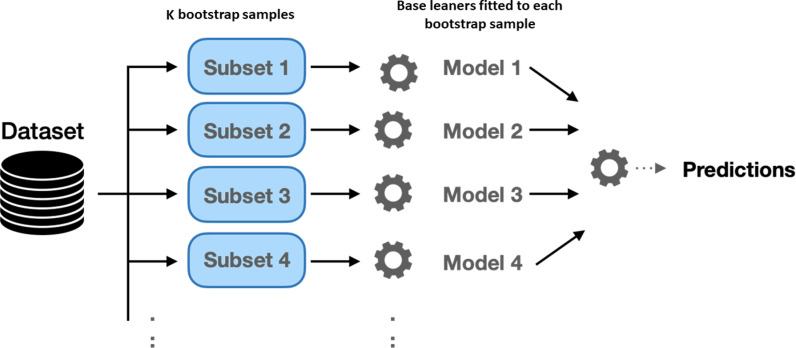
Bagging consists of fitting several base models on different bootstrap samples and building an ensemble model that averages the results of these base learners.

By averaging the predictions, bagging effectively reduces the variance associated with individual models, leading to more reliable and generalized predictions [12], as shown in Figure 1. Bagging is particularly effective with high-variance, low-bias models such as decision trees, making it a popular choice in ensemble regression frameworks.

Here, we extend the traditional bagging approach by developing a heterogeneous bagging algorithm. Unlike homogeneous bagging, where all base learners are of the same type, heterogeneous bagging incorporates diverse types of base models (e.g., decision trees, support vector machines, neural networks) within the ensemble. This diversity can further enhance the ensemble’s performance by capturing a wider range of data patterns and reducing the risk of model-specific biases. The heterogeneous bagging algorithm developed for this work serves as a benchmark for comparing the effectiveness of the proposed RRMSE Voting Regressor.

#### 2.1.2 Voting regression.

Voting regression is another widely used averaging ensemble method. Unlike bagging, which typically uses identical base learners trained on different subsets of data, voting regression combines predictions from different types of regression models trained on the entire dataset. This method leverages the unique strengths of each base model to improve overall prediction accuracy.

In voting regression, each base model makes a prediction for the target variable, and these predictions are then combined, usually by averaging, to produce the final ensemble prediction. The way these predictions are combined is crucial, as it directly influences the accuracy and reliability of the final output.

**Fig 2:**
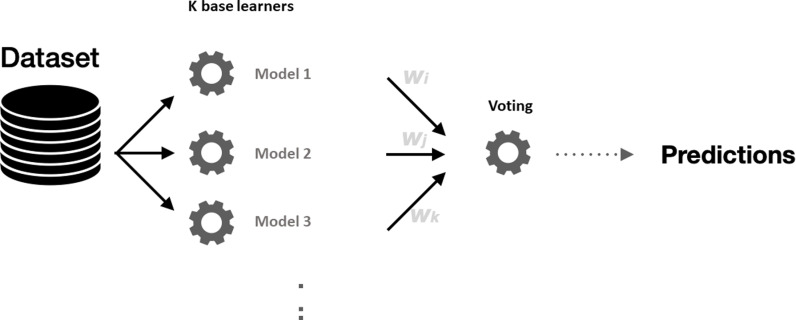
Voting regression involves fitting several base models on the entire dataset and building an ensemble model that averages the results of these base learners, with each base learner assigned its own weight.

Figure 2 illustrates the structure of a voting regression model. Each base learner is assigned a specific weight, which determines its influence on the final prediction. Properly assigning these weights is essential for maximizing the ensemble’s performance, as it ensures that more accurate models have a greater impact on the final prediction. Here, we focus on developing a dynamic weighting function for heterogeneous ensembles, allowing for more flexible and accurate combination of diverse base learners. This approach contrasts with traditional voting regressors that typically use uniform weights, which may not fully exploit the potential of each base model’s strengths.

### 2.2 Weighting functions

The effectiveness of ensemble methods largely depends on how the predictions of base learners are combined. Weighted averaging introduces a mechanism to assign different weights to base learners based on their performance, thereby enhancing the ensemble’s overall predictive power. This section reviews related work on weighting functions used for ensemble integration. Equation 4 shows that ensemble integration is performed using a linear combination of the base learners’ predictions:


$$\hat{f_{f}}\left(x\right)=\overset{k}{\underset{i=1}{\sum }}h_{i}\left(x\right)\cdot \hat{f_{i}}\left(x\right)$$ (4)

Here, *h*_*i*_ ( *x* )  represents the weighting function assigned to the *i* -th base learner’s prediction. Weighting functions can be categorized into two types: constant weighting functions, where *h*_*i*_ ( *x* )  are fixed coefficients, and non-constant weighting functions, where the weights vary depending on the input features *x*.

#### 2.2.1 Constant weighting functions.

Constant weighting functions assign the same set of coefficients to base learners, regardless of the input data. This approach simplifies the integration process but may not fully leverage the individual strengths of each base model [13]. For example, the Basic Ensemble Method (BEM) proposed by [14] uses equal weights for all base learners:


$$\hat{f_{BEM}}\left(x\right)=f\left(x\right)-\frac{1}{k}\overset{k}{\underset{i=1}{\sum }}m_{i}\left(x\right)\;,$$ (5)

where


$$m_{i}\left(x\right)=f\left(x\right)-\hat{f_{i}}\left(x\right)\;.$$ (6)

BEM assumes that the errors *m*_*i*_ ( *x* )  are independent and have zero mean. To address potential issues with multi-collinearity, [14] modified BEM to create the Generalized Ensemble Method (GEM):


$$\hat{f_{GEM}}\left(x\right)=\overset{k}{\underset{i=1}{\sum }}\left[{\text{α}}_{i}\cdot \hat{f_{i}}\left(x\right)\right]=f\left(x\right)+\overset{k}{\underset{i=1}{\sum }}\left[{\text{α}}_{i}\cdot m_{i}\left(x\right)\right]\;,$$ (7)

where


$$\overset{k}{\underset{j=1}{\sum }}{\text{α}}_{j}=1,$$

$${\text{α}}_{i}=\frac{\sum_{j=1}^{k}C_{ij}^{-1}}{\sum_{l=1}^{k}\sum_{j=1}^{k}C_{ij}^{-1}},$$ (8)

$$C_{ij}=E\left[m_{i}\left(x\right)\cdot m_{j}\left(x\right)\right].$$

However, GEM faces challenges such as multi-collinearity, which leads to wide confidence intervals for the coefficients ${\text{α}}_{\text{i}}$ due to the necessity of calculating the inverse matrix $C^{-1}$. To mitigatthis issue, [15] integrated the ensemble integration phase into the ensemble selection phase. By selecting models from a pool to include in the ensemble and using simple averaging as the weighting function, the coefficients ${\text{α}}_{i}$ are implicitly determined based on the frequency each model is selected. Another approach by [16] utilizes Principal Component Regression (PCR) to address multi-collinearity. PCR determines principal components and selects the number of components to use based on the amount of variance they explain, thereby reducing the complexity and variance of the coefficient estimates.

#### 2.2.2 Non-constant weighting functions.

Non-constant weighting functions assign different weights to base learners based on the input features *x*, allowing the ensemble to adapt its predictions dynamically. Unlike constant weighting, non-constant weighting can capture more nuanced relationships between the input data and model performance [17].

Static weighting functions use predefined coefficients based on prior domain knowledge or expertise, which do not change with the input data. In contrast, dynamic weighting functions determine the weights *h*_*i*_ ( *x* )  based on the performance of base learners for specific input instances. This allows the ensemble to prioritize models that perform better on similar data points [18].

For instance, [19] and [20] employ k-nearest neighbors with Euclidean distance and weighted k-nearest neighbors to select predictors dynamically. Dynamic weighting [21] assigns weights to each base learner based on their localized performance within a group of k-nearest neighbors. The final prediction is then computed as a weighted average of the predictions from these selected models. Here, we implemented a Dynamic Weighting Voting Regressor (DWR) to compare its performance with the proposed RRMSE Voting Regressor. By dynamically assigning weights based on model performance in specific regions of the input space, DWR serves as a relevant baseline for evaluating the effectiveness of our RRMSE-based weighting approach.

In summary, ensemble learning for regression encompasses a variety of methods aimed at improving prediction accuracy and robustness by combining multiple base learners. Averaging methods, such as bagging and voting regression, offer straightforward approaches to reducing model variance through parallel training and prediction aggregation. Boosting methods, although not the focus of this study, provide alternative strategies for sequentially enhancing model performance by addressing residual errors. Weighted averaging introduces a critical enhancement by allowing differential influence of base learners based on their performance, which is especially beneficial in heterogeneous ensembles where diversity among base models is leveraged for improved predictions.

## 3. RRMSE voting regressor

Relative Root Mean Squared Error (RRMSE) extends the Root Mean Squared Error (RMSE) by normalizing it with the scale of the actual values. This normalization facilitates a relative comparison of errors across different models and datasets. RMSE is defined as:


$$\text{RMSE}\left(f,\hat{f}\right)=\sqrt{\frac{1}{n}\overset{n}{\underset{i=1}{\sum }}{\left(f_{i}-\hat{f_{i}}\right)}^{2}}$$ (9)

where:

- $\hat{{\text{f}}_{\text{i}}}$ is the predicted value for the *i* -th sample,
- *f*_*i*_ is the actual value for the *i* -th sample,
- *n* is the total number of samples.

To account for the scale of the target variable, RRMSE is defined as:


$$\text{RRMSE}\left(f,\hat{f}\right)=\sqrt{\frac{\frac{1}{n}\sum_{i=1}^{n}{\left(f_{i}-\hat{f_{i}}\right)}^{2}}{\sum_{i=1}^{n}f_{i}^{2}}}$$ (10)

RRMSE provides a dimensionless measure of error, enabling fair comparisons between models operating on different scales. A well-performing ensemble comprises accurate predictors with errors distributed across various regions of the input space. By decomposing the generalization error into different components, RRMSE serves as a dynamic weighting function to optimize ensemble integration.

The RRMSE Voting Regressor assigns weights to base learners based on their RRMSE values, ensuring that more accurate models have a greater influence on the final prediction. The algorithm consists of four main phases: initialization, RRMSE calculation, weight assignment, and ensemble prediction, as shown in Algorithm 1.

The algorithm operates through four main phases. In the first phase, a constant *ϵ* is calculated to prevent division by zero during weight assignment. This constant is derived as the average absolute deviation of the training labels from their mean:


$$\text{avg}=\frac{1}{n}\overset{n}{\underset{i=1}{\sum }}y_{i}$$

$$\text{sum}=\overset{n}{\underset{i=1}{\sum }}\left|y_{i}-\text{avg}\right|$$

$$\epsilon =\frac{\text{sum}}{n}$$

During the second phase, the RRMSE for each base learner is computed using Equation 10. These RRMSE values are stored in an array for subsequent weight calculation. In the third phase, weights *W*_*k*_ are assigned to each base learner inversely proportional to their RRMSE values:


$$W_{k}=\frac{\frac{1}{{\text{RRMSE}}_{k}+\epsilon }}{\sum_{j=1}^{k}\frac{1}{{\text{RRMSE}}_{j}+\epsilon }}$$

This normalization ensures that the sum of all weights equals one, allowing for a balanced contribution from each base learner based on their relative performance.

Finally, in the fourth phase, ensemble predictions are generated by computing the weighted sum of the base learners’ predictions for each test sample:


$$\hat{y}\left(x\right)=\overset{K}{\underset{k=1}{\sum }}W_{k}\cdot \hat{f_{k}}\left(x\right)$$

This weighted averaging integrates the strengths of individual base learners, enhancing the overall accuracy and reliability of the ensemble. Using RRMSE as a weighting function ensures that the influence of each base learner is proportional to its relative error. By normalizing RMSE, RRMSE accounts for the scale of the target variable, making the weighting mechanism robust across different datasets. The inverse proportionality of weights to RRMSE emphasizes models with lower relative errors, thereby optimizing the ensemble’s predictive performance. Moreover, the normalization step in weight assignment ensures that the sum of all weights equals one, maintaining a balanced contribution from each base learner. This approach mitigates the risk of any single model disproportionately dominating the ensemble, promoting a more harmonious integration of diverse models.

## 4. Experiments

To evaluate the effectiveness of the RRMSE Voting Regressor, we conducted experiments using six well-known regression datasets, as listed in Table 1. These datasets vary in domain, size, and feature count, providing a comprehensive assessment of the proposed method. Specifically, the Abalone dataset [22] comprises 4,177 instances with 8 physical measurements, aiming to predict the age of Abalone shells. The Car dataset [22] includes 398 instances and 7 features, with the target variable being fuel consumption measured in miles per gallon (mpg). The Diamond dataset contains 53,940 instances and 9 attributes, focusing on predicting diamond prices. The Airfoil dataset [22] consists of 1,503 instances with 5 features, used to predict the scaled sound pressure level based on aerodynamic and acoustic measurements. The Smart Grid Stability dataset [22] includes 60,000 instances and 12 features, relating to the local stability analysis of a 4-node star system implementing decentralized smart grid control. Lastly, the Elongation dataset contains 385 instances with 17 chemical components as input attributes, aimed at predicting the elongation of steel.

**Table 1:** Datasets used in experiments and their properties.

Dataset	Domain	Instances	Features
Abalone	Zoology	4,177	8
Car	Automobile	398	7
Diamond	Consumption	53,940	9
Airfoil	Aerodynamics	1,503	5
Smart Grid Stability	Electricity	60,000	12
Elongation	Metallurgy	385	17

To benchmark the performance of the RRMSE Voting Regressor, three state-of-the-art ensemble regression algorithms are also evaluated. The first baseline is the Voting Regressor with uniform weights (VRU), implemented using the Scikit-Learn library [23]. The second baseline is the Bagging Regressor with uniform weights (BR), which extends the traditional bagging approach to a heterogeneous ensemble. The third baseline is the DWR [21], which assigns weights to base learners based on their localized performance. While VRU is readily available in Scikit-Learn, both BR and DWR are implemented by the author in Python to ensure a fair comparison with the proposed method.

Model performance was assessed using multiple evaluation metrics to provide a comprehensive understanding of each algorithm’s predictive capabilities. In addition to RMSE (cf. Equation 9), the following metrics are employed:


$$\text{MAE}\left(f,\hat{f}\right)=\frac{1}{n}\overset{n}{\underset{i=1}{\sum }}\left|f_{i}-\hat{f_{i}}\right|$$ (11)

Mean Absolute Error (MAE) measures the average magnitude of errors without considering their direction. It is calculated as the average of the absolute differences between predicted and actual values. Mean Squared Error (MSE) is another widely used loss metric that penalizes larger errors more heavily than smaller ones. It is defined as the average of the squared differences between predicted and actual values.


$$\text{MSE}\left(f,\hat{f}\right)=\frac{1}{n}\overset{n}{\underset{i=1}{\sum }}{\left(f_{i}-\hat{f_{i}}\right)}^{2}$$ (12)

The $R^{2}$ score, or coefficient of determination, represents the proportion of variance in the target variable that is explained by the model. It provides an indication of the model’s goodness of fit, with higher values indicating better explanatory power.


$$R^{2}\left(f,\hat{f}\right)=1-\frac{\sum_{i=1}^{n}{\left(f_{i}-\hat{f_{i}}\right)}^{2}}{\sum_{i=1}^{n}{\left(f_{i}-\bar{f}\right)}^{2}}$$

For each ensemble algorithm, the same set of base learners was utilized to ensure consistency in comparison. These base learners include Linear Regression (LR), K-Nearest Neighbors Regression (KNN), Stochastic Gradient Descent Regression (SGD), and Random Forest Regression (RF). All four regression models are implemented using the Scikit-Learn library [23]. The datasets are split into training and testing sets using an 80%-20% ratio, respectively. This split allows sufficient data for training while reserving a representative portion for unbiased evaluation of model performance.

## 5. Results

The performance of the RRMSE Voting Regressor was evaluated and compared against three other ensemble methods: VRU, BR, and DWR. All base learners were configured with default hyperparameters to ensure a fair comparison. Table 2 presents the results across four regression metrics — MAE, MSE, RMSE, and $R^{2}$ score — for each of the six testing datasets. The numbers in parentheses indicate the relative ranking of each algorithm for a specific metric and dataset, where a lower rank signifies better performance.

**Table 2:** Performance of different algorithms across various regression metrics and datasets.

Dataset	Methods	MAE	MSE	RMSE	$R^{2}$
Abalone	RRMSE (2)	1.5102 (2)	4.7140 (2)	2.1712 (2)	0.5645 (2)
	BR (3)	1.5290 (3)	4.7995 (3)	2.1908 (3)	0.5566 (3)
	VRU (1)	1.4949 (1)	4.6174 (1)	2.1488 (1)	0.5735 (1)
	DWR (4)	1.5853 (4)	4.8962 (4)	2.2127 (4)	0.5477 (4)
Car	RRMSE (1)	1.6758 (1)	4.7219 (1)	2.1730 (1)	0.9122 (1)
	BR (3)	1.9065 (3)	6.0156 (3)	2.4527 (3)	0.8881 (3)
	VRU (2)	1.8426 (2)	5.4789 (2)	2.3407 (2)	0.8981 (2)
	DWR (4)	2.4460 (4)	9.6812 (4)	3.1115 (4)	0.8199 (4)
Diamond	RRMSE (1)	301.8838 (1)	330903 (1)	575.24 (1)	0.9792 (1)
	BR (3)	449.6199 (3)	615979 (3)	784.65 (3)	0.9613 (3)
	VRU (2)	437.4929 (2)	571903 (2)	756.24 (2)	0.9640 (2)
	DWR (4)	718.8416 (4)	1473423 (4)	1213.84 (4)	0.9073 (4)
Airfoil	RRMSE (1)	1.8090 (1)	5.3482 (1)	2.3126 (1)	0.8932 (1)
	BR (3)	3.1896 (3)	16.0696 (3)	4.0074 (3)	0.6794 (3)
	VRU (2)	3.0898 (2)	14.9672 (2)	3.8688 (2)	0.5414 (4)
	DWR (4)	3.6852 (4)	22.9774 (4)	4.7935 (4)	0.8199 (4)
Smart Grid Stability	RRMSE (1)	0.0075 (1)	0.000095 (1)	0.0098 (1)	0.9287 (1)
	BR (3)	0.0116 (3)	0.0002 (3)	0.0146 (3)	0.8414 (3)
	VRU (2)	0.0112 (2)	0.0001 (2)	0.0140 (2)	0.8541 (2)
	DWR (4)	0.0174 (4)	0.0004 (4)	0.0219 (4)	0.6425 (4)
Elongation	RRMSE (1)	0.8219 (1)	1.1775 (1)	1.0851 (1)	0.6271 (1)
	BR (3)	0.8524 (2)	1.3431 (3)	1.1589 (3)	0.5746 (3)
	VRU (2)	0.8531 (3)	1.3174 (2)	1.1478 (2)	0.5828 (2)
	DWR (4)	0.9984 (4)	1.6482 (4)	1.2838 (4)	0.4780 (4)

Numbers in parentheses indicate the relative ranking of each algorithm for that specific metric and dataset, with lower ranks representing better performance.

Table 2 reveals that the RRMSE Voting Regressor outperforms the other three algorithms in five out of six datasets across most metrics. The exception is the Abalone dataset, where RRMSE achieves the second-best average rank. This indicates that while RRMSE generally excels, there are specific instances where other methods like VRU may perform comparably or better.

To determine the statistical significance of these performance differences, we employed the Friedman Aligned Rank Test [24], which is suitable for comparing multiple algorithms across multiple datasets. The test confirmed that there are significant differences among the algorithms at a significance level of α=0.05. Subsequently, pairwise post-hoc Friedman Aligned Rank Tests were conducted to identify which specific algorithm pairs differed significantly. The results are detailed in Table 3.

**Table 3:** Pairwise comparisons and significance tests between algorithms for each regression metric.

MAE	RRMSE	BR	VRU	DWR
RRMSE	–	6/0/0	5/0/1	6/0/0
BR	0.2529	–	1/5/0	6/0/0
VRU	0.4142	0.7439	–	6/0/0
DWR	*** 0.0011	** 0.0337	** 0.0143	–
**MSE**				
RRMSE	–	6/0/0	5/0/1	6/0/0
BR	0.1779	–	0/6/0	6/0/0
VRU	0.4379	0.5676	–	6/0/0
DWR	*** 0.0010	** 0.0550	** 0.0127	–
**MAE**				
RRMSE	–	6/0/0	5/0/1	6/0/0
BR	0.1279	–	0/6/0	6/0/0
VRU	0.3379	0.4673	–	6/0/0
DWR	*** 0.0010	** 0.0561	** 0.0137	–
**MAE**				
RRMSE	–	6/0/0	5/0/1	6/0/0
BR	** 0.04122	–	0/6/0	6/0/0
VRU	0.2884	0.3271	–	6/0/0
DWR	*** 0.00008	** 0.0603	*** 0.00426	–

Upper diagonal cells show the win/lose/tie counts, while the lower diagonal cells present the p-values from the post-hoc Friedman Aligned Rank Tests. Asterisks indicate significant differences: *  for α=0.10, ** for α=0.05, and *** for α=0.01

Table 3 provides a detailed comparison between each pair of algorithms for every regression metric. The upper diagonal section displays the number of datasets where one algorithm outperforms, underperforms, or ties with another. For instance, under the MAE metric, the RRMSE Voting Regressor wins against both BR and DWR in all six datasets, ties with VRU once, and does not lose in any. The lower diagonal section presents the p-values from the post-hoc Friedman Aligned Rank Tests, indicating whether the performance differences are statistically significant.

The experimental results demonstrate that the RRMSE Voting Regressor consistently outperforms the BR and DWR across multiple datasets and evaluation metrics. Specifically, RRMSE achieves the best average rank in five out of six datasets, indicating its robust ability to minimize prediction errors and explain variance effectively.

In the Abalone dataset, RRMSE ranks second, closely trailing VRU. This suggests that while RRMSE generally excels, there are scenarios where traditional ensemble methods like VRU can perform equally well or better. However, in the Car, Diamond, Airfoil, and Smart Grid Stability datasets, RRMSE maintains its top position, highlighting its versatility and effectiveness across diverse domains. The Elongation dataset further reinforces RRMSE’s superiority, where it achieves the best performance across all metrics, followed by BR, VRU, and DWR. The statistical significance tests confirm that these performance differences are not merely due to chance, particularly the significant improvements over DWR and BR.

The Diamond and Smart Grid Stability datasets, with their large and varied feature sets, highlight RRMSE’s capability to handle complex regression tasks effectively. The high ${\text{R}}^{2}$ scores across these datasets indicate that RRMSE not only reduces errors but also explains a substantial portion of the variance in the target variables. While VRU shows competitive performance, especially in the Abalone and Car datasets, the lack of statistically significant improvements over RRMSE suggests that RRMSE’s dynamic weighting provides a consistent advantage without being outperformed in specific instances.

Overall, the RRMSE Voting Regressor proves to be a robust and effective ensemble method for regression tasks, offering significant improvements over traditional and dynamic weighting approaches. Its ability to dynamically adjust weights based on relative errors ensures that more accurate models contribute more significantly to the final prediction, thereby enhancing the ensemble’s overall performance and reliability.

## 6. Conclusions

We introduce a novel weighted voting ensemble algorithm for regression tasks, named the RRMSE Voting Regressor. The algorithm assigns weights to base learners based on the inverse of their RRMSE, ensuring that more accurate models have a greater influence on the final prediction.

The experimental results presented in Section 5 demonstrate that the RRMSE Voting Regressor significantly outperforms the BR and DWR across multiple datasets and evaluation metrics. While the RRMSE Voting Regressor also shows improved performance compared to the VRU, the enhancement is marginal, indicating that VRU remains a competitive baseline in certain scenarios.

These findings confirm that the RRMSE Voting Regressor effectively enhances ensemble regression performance by dynamically weighting base learners according to their predictive accuracy. Moving forward, it would be valuable to further explore and refine the RRMSE Voting Regressor to enhance its predictive capabilities. Potential avenues for improvement include experimenting with a wider variety of base learners and optimizing their hyperparameters using techniques such as Random Search or Bayesian Optimization. Additionally, integrating more sophisticated base learner selection strategies could further elevate the ensemble’s performance.

In conclusion, the RRMSE Voting Regressor represents a promising advancement in ensemble regression methodologies, offering a robust framework for leveraging the strengths of individual models to achieve superior predictive accuracy. Future research aimed at optimizing base learner selection and hyperparameter tuning holds the potential to unlock even greater performance gains.
